# Smartphones for Smarter Delivery of Mental Health Programs: A Systematic Review

**DOI:** 10.2196/jmir.2791

**Published:** 2013-11-15

**Authors:** Tara Donker, Katherine Petrie, Judy Proudfoot, Janine Clarke, Mary-Rose Birch, Helen Christensen

**Affiliations:** ^1^Black Dog InstituteUniversity of New South WalesSydneyAustralia

**Keywords:** mobile applications, mobile mental health, mobile phones, self-help, depression, anxiety, stress, substance use

## Abstract

**Background:**

The rapid growth in the use of mobile phone applications (apps) provides the opportunity to increase access to evidence-based mental health care.

**Objective:**

Our goal was to systematically review the research evidence supporting the efficacy of mental health apps for mobile devices (such as smartphones and tablets) for all ages.

**Methods:**

A comprehensive literature search (2008-2013) in MEDLINE, Embase, the Cochrane Central Register of Controlled Trials, PsycINFO, PsycTESTS, Compendex, and Inspec was conducted. We included trials that examined the effects of mental health apps (for depression, anxiety, substance use, sleep disturbances, suicidal behavior, self-harm, psychotic disorders, eating disorders, stress, and gambling) delivered on mobile devices with a pre- to posttest design or compared with a control group. The control group could consist of wait list, treatment-as-usual, or another recognized treatment.

**Results:**

In total, 5464 abstracts were identified. Of those, 8 papers describing 5 apps targeting depression, anxiety, and substance abuse met the inclusion criteria. Four apps provided support from a mental health professional. Results showed significant reductions in depression, stress, and substance use. Within-group and between-group intention-to-treat effect sizes ranged from 0.29-2.28 and 0.01-0.48 at posttest and follow-up, respectively.

**Conclusions:**

Mental health apps have the potential to be effective and may significantly improve treatment accessibility. However, the majority of apps that are currently available lack scientific evidence about their efficacy. The public needs to be educated on how to identify the few evidence-based mental health apps available in the public domain to date. Further rigorous research is required to develop and test evidence-based programs. Given the small number of studies and participants included in this review, the high risk of bias, and unknown efficacy of long-term follow-up, current findings should be interpreted with caution, pending replication. Two of the 5 evidence-based mental health apps are currently commercially available in app stores.

## Introduction

Global mobile phone penetration reached 91% at the end of 2012, with 4.3 billion unique mobile subscribers [1] identified. Mobile health (mHealth)—specifically, mental health supported by mobile devices—thus has the potential to be delivered to large numbers of people worldwide. The first mobile software applications or “apps” became available to download on a mobile device in 2008. Since then, penetration has increased rapidly and is anticipated to continue rising. As of September 2012, an estimated 1,520,000 apps had been developed for mobile devices [2], and around 13,600 health apps intended for use by consumers were available for download in Apple’s App Store [3]. About 6% of these apps targeted mental health outcomes, while 18% focused on related health issues, such as sleep, stress, relaxation, and smoking behaviors. A survey among the Australian general public indicated that 76% would be interested in using mobile phones for mental health monitoring and self-management [4]. This suggests that mHealth is acceptable and may be a useful vehicle for enhancing access to evidence-based monitoring and self-help for individuals with mild-to-moderate common mental health conditions [4]. Clinical practice guidelines recommend cognitive behavior therapy (CBT) and self-help resources (such as mHealth) as options for psychological treatment for individuals experiencing mild-to-moderate symptoms of anxiety or depression [5]. mHealth apps can be used as stand-alone self-help programs or as a conjunctive treatment modality in guided programs, for example, part of a website or through direct contact with a mental health professional. The app can include treatment components such as cognitive therapy (CT), behavioral activation (BA), psychoeducation, or monitoring of symptoms.

Advantages of mHealth include the improvement of treatment accessibility and participant retention, real-time symptom and activity monitoring and tracking of treatment progress through ecological momentary assessment (EMA), provision of personalized feedback and motivational support, portability and flexibility of use, and the potential to improve adherence to treatment [6-10]. However, there are also disadvantages with using mobile devices for mental health. Technical problems and factors related to telecommunication can arise (eg, battery failures, reliability and sustainability of connections [11]), and issues of data security, patient privacy, and the identification and timely management of crises and risk of harm must be carefully considered when integrating smartphone technology into behavioral health care [12].

Previous research suggests that mental health interventions delivered through mobile apps can be effective in treating a range of mental health disorders, such as depression, stress, anxiety, and smoking cessation [6,7]. However, the thriving development of mental health apps warrants a systematic review of the available evidence base in this growing area. Previous reviews examining evidence-based mental health apps did not incorporate quantitative analyses [12] or included mHealth interventions that were not directly downloadable as an app (such as programs using SMS [short message service] text messaging or Internet-enabled interventions on mobile phones [6,13]). Therefore, the aim of this paper is to systematically review the available evidence-based apps directly downloadable on mobile devices (such as smartphones and tablets) for mental health symptoms or disorders (depression, anxiety, substance use, sleep disorders, suicidal behavior, psychotic disorders, eating disorders, stress, gambling) in children, adolescents, adults, and older individuals.

## Methods

### Search Strategy and Selection of Studies

A comprehensive literature search in bibliographic databases (MEDLINE, Embase, the Cochrane Central Register of Controlled Trials, PsycINFO, PsycTESTS, and Compendex and Inspec) for relevant articles published from January 1, 2008 (launch date of the first app), to May 30, 2013, was conducted. Terms indicative of mobile apps and mental health disorders were used to search these databases, with the search being limited to “humans”, English, and peer-reviewed journals (see Multimedia Appendices 1-3 for the full search string). The identified titles and abstracts were screened for eligibility by 2 independent researchers. Full text copies of all potentially relevant papers, or papers where there was insufficient information in the abstract to determine eligibility, were obtained. Full text articles were further screened and discarded from further analyses if they met exclusion criteria. In addition, references of earlier reviews and reference lists of the included primary articles were examined. Furthermore, key technology journals (Cybertechnology, Behavior and Social Networking; Journal of Medical Internet Research; and Studies in Health Technology and Informatics) were hand-searched. We also reviewed Beacon, a website for evidence-based online programs for mental health, developed and delivered by the Centre for Mental Health Research at the Australian National University. Finally, a search was conducted of prominent individual authors’ and researchers’ names in the field of mHealth or Internet interventions (see Multimedia Appendix 4) in MEDLINE. Data extraction of relevant articles was completed by 2 independent researchers, with disagreements resolved through discussion or with a third researcher.

We applied strict inclusion criteria in order to investigate any evidence-based mental health apps that could be downloaded from app stores (eg, Google Play for Google Android [14] or the Apple iTunes store [15]). Studies examining the effects of mental health apps on mental health symptoms or disorders (depression, anxiety, substance use, sleep disorders, suicidal behavior, self-harm, psychotic disorders, eating disorders, stress, and gambling) that were directly downloadable on a mobile device (eg, smartphone or tablet) compared with a control group were included. The control group could consist of a wait list, treatment-as-usual, or another treatment. Studies without a control group (pre-post design) were also included. There was no restriction on participant age. Studies were excluded if they did not include an intervention or if mental health symptoms/disorders were not an outcome, and if the intervention was an Internet-based intervention, virtual reality exposure treatment, interactive voice response technology intervention, or a text messaging-only intervention without a mobile application component*.* Studies were also excluded if the intervention was downloaded on a computer and transferred (eg, through Bluetooth or infrared) to a mobile device, if the intervention targeted a medical disorder (eg, irritable bowel syndrome, diabetes), if the paper provided a description of the mobile application but no outcome data, and if the intervention was developed before 2008. Conference abstracts, protocol papers, case studies, non-peer reviewed papers, and non-English papers were also excluded.

### Quality Assessment

Study quality was assessed according to 6 basic criteria of the Cochrane Risk of Bias Assessment Tool [16]: sequence generation, allocation concealment, blinding of outcome assessors, incomplete outcome data, selective outcome reporting, and other sources of bias. For the third criterion (blinding of outcome), we omitted blinding of participants since blinding participants for treatment allocation is rarely achievable in intervention trials for mental health disorders.

### Outcome Measures

Primary outcome measures included reduction of depression symptoms, anxiety symptoms, substance use, sleep disturbance, suicidal behavior (suicide ideation, suicide plans, and attempts), self-harm, psychotic symptoms, symptoms of eating disorders, and gambling, as assessed with validated mental health scales.

### Statistical Analyses

When data were available and extractable, intention-to-treat (ITT) within-group and between-group effect sizes (Cohen’s *d*) for the intervention group were calculated by taking the difference between the mean pre- and posttest scores (within-group effect size) or the difference of the posttest scores (between-group effect size) and dividing by the pooled standard deviation. Effect sizes of 0.8 can be assumed to be large, while effect sizes of 0.5 are moderate, and effect sizes of 0.2 are small [17]. Where authors provided only *t* test statistics, we computed effect sizes using the formula: *d*=*t* / sort(*df*) [18]. Hedges’ *g* effect sizes were converted to Cohen’s *d.* Authors were contacted to provide additional data if needed. Two studies [19,20] did not provide sufficient data to calculate ITT within-group effect sizes.

## Results

### Selection and Inclusion of Studies

**Figure 1:**
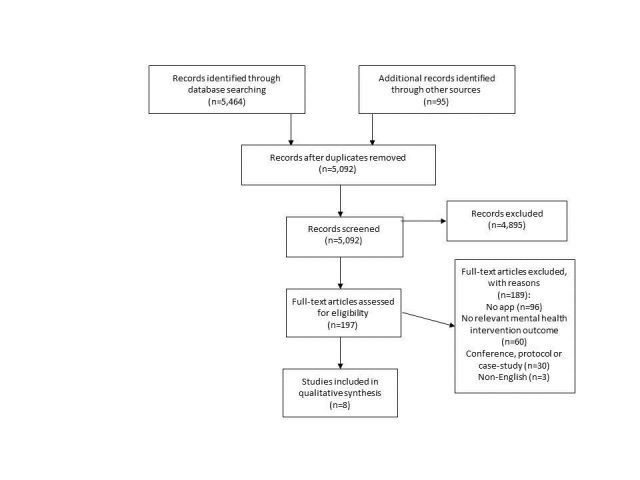
Flow diagram of participants.

A total of 5464 abstracts in MEDLINE (n=1859), Embase (n=1030), the Cochrane Central Register of Controlled Trials (n=277), PsycINFO (n=1095), PsycTESTS (n=1), and Compendex and Inspec (n=1203) were examined (N=4997 abstracts in total, after removal of duplicates). The majority of records that were excluded addressed nonpsychological technical issues, provided descriptions of mobile apps without outcome data, or were protocol papers or conference abstracts. Of these, 133 full text papers potentially eligible for inclusion were retrieved for further consideration, of which 126 were excluded. Seven trials met inclusion criteria. A further screening for potentially relevant references in recent systematic reviews or meta-analyses and the included studies, individual author names in MEDLINE, and hand-searching of technology journals (Cybertechnology, Behavior and Social Networking; Journal of Medical Internet Research; and Studies in Health Technology and Informatics [January 1, 2008, to May 30, 2013]) and the Beacon website resulted in 95 potentially relevant abstracts and retrieval of 64 additional full text papers for further assessment. Of these, only 1 study met inclusion criteria and was included in the final analysis. In total, 8 trials were identified. These described 5 apps (Mobilyze! [11], mobiletype [21,22], DBT Coach [23], Mobile Stress Management [19,20,24], and Get Happy Program [25]) (see Figure 1 for a flowchart of the screening process). There was a high degree of consensus among raters who screened the titles and abstracts (an interrater reliability of 95.2%).

### Characteristics of Included Studies

A total of 227 participants were recruited across all studies. One study [19] did not provide sufficient information about sample size per treatment arm. Of the 8 included studies, 4 trials describing 3 apps assessed depression (Mobilyze!, mobiletype, Get Happy Program), and 3 studies describing 1 app (Mobile Stress Management) assessed stress as a primary outcome measure. Substance use was used as an outcome measure in 1 study (DBT Coach).

Table 1 provides an overview of the included studies (see Multimedia Appendix 5 for the complete version of the table). One study used BA and another used CBT as the therapeutic mode of the intervention. Two studies described a trial delivering emotional self-awareness (ESA), 1 study was based on dialectical behavioral therapy (DBT) and opposite action (ie, emotional regulation skills), and 3 studies described an app delivering stress inoculation training (SIT) as the content of the intervention. Four studies describing 3 trials used an attention-placebo as a control group, 1 study used an active comparison, and 1 study did not specify the nature of the control group. Two studies used a pre-post design without a control group, and all studies except one were feasibility and/or pilot studies. Two studies recruited adults from the community, 1 study recruited from an outpatient clinic, and 2 studies recruited from the workplace. Two studies describing 1 trial recruited adolescents from general practice, and 1 study targeted female university students. Four studies delivered the intervention through a stand-alone mobile app, while 3 studies describing 2 trials used a mobile app alongside a website and EMA to deliver the intervention. One study used a mobile application in conjunction with traditional face-to-face therapy. All included studies delivered the program on a mobile phone, with 1 study also including iPads. Delivery length varied between 6 days and 8 weeks. Five studies assessed posttest outcomes only, whereas 3 studies describing 2 trials undertook follow-up assessments as well (6 weeks and 3 months). Five studies describing 4 apps were guided by mental health professionals through phone or email contact, whereas in 3 studies describing 1 app, participants independently navigated their way through the trial.

**Table 1:** Psychosocial studies of applications on mobile devices (Intention-To-Treat). App: Application; BA: Behavioral Activation; BCQ: Behavior Confidence Questionnaire; BDI: Beck Depression Inventory; BPD: Borderline Personality Disorder; BSI: Brief Symptom Inventory; COPE: COPE Inventory; DASS: Depression, Anxiety, and Stress Scale; DBT: Dialectical Behavior Therapy; ESA: Emotional Self Awareness; F2F: Face-to-Face; GAD-7: Generalized Anxiety Disorder-7 item scale; GP: General Practitioner; It: Italian; K10: Kessler Psychological Distress Scale-10 item scale; MDD: Major Depressive Disorder; MHP: Mental Health Professional (psychologist or psychotherapist, GP); MINI: Mini-International Neuropsychiatric Interview; NA: Not Applicable; OA: Opposite Action; PHQ: Patient Health Questionnaire; QIDS-C: Quick Inventory of Depression Symptoms-Clinician Rated; RCT: Randomized Controlled Trial; SIT: Stress Inoculation Training; STAI: State-Trait Anxiety Inventory.

Author (year); name of app	Trial	Primary outcome measure	Study sample	Intervention group	Control group	Delivery type	Delivery length and support	Within^f^ and between^g^ effect size (Cohen’s *d*)
Burns et al (2011); *Mobilyze!*	Pre-post pilot	MDD	Adults from the com-munity	n=8; BA	NA	Mobile app + website + EMA on mobile phone	8 weeks; MHP	PHQ-9: *d*=1.95^a,e,f^ *d*=2.28^a,e,f^ GAD-7: *d*=1.37^a,e,f^
Kauer et al (2012); Reid et al (2011); *Mobile-type*	RCT	MDD	Adolescents from general practice	n=68; ESA + Individualized data summary reports + meeting with GP	n=49; Attention control + part of the individualized data summary reports + meeting with GP	Stand-alone mobile app + EMA on mobile phone	8 modules over 2-4 weeks; MHP	DASS Stress: *d*=0.37^a,f^ *d*=0.59^b,e,f^ *d*=0.14^a,g^ *d*=0.22^b,g^ DASS Anxiety: *d*=0.31^b,f^ *d*=0.45^b,d,f^ *d*=0.25^a,g^ *d*=0.07^b,g^ DASS Depression: *d=*0.34 *d*=0.64^b,e^ *d*=0.11^a,g^ *d*=0.09^b,g^ ESA: *d*=0.31^a^ *d*=0.66^b,d^ *d*=0.09^a,e,g^ *d*=0.58^b,d,g^
Rizvi et al (2011); *DBT Coach*	Pre-post pilot	BPD and substance use	Adults from out-patient clinic	N=21; DBT + OA	NA	Mobile app on mobile phone + F2F DBT	10-14 days; MHP	BDI: *d*=0.55^a,d,f^ BSI: *d*=0.43^a,d,f^ BCQ: *d*=0.59^a,d,f^ Emotional intensity to use substance^:^ *d*=0.52^c,d,f^ Urge to use substance: *d*=0.29^c,d,f^
Villani et al (2012); *Mobile Stress Management*	RCT	Stress	Female oncology nurses	n=8; SIT	n=8; Attention control	Stand-alone mobile app on mobile phone	8 videos over 4 weeks; no support	NA
Villani et al (2011); *Mobile Stress Management*	RCT	Stress	Female oncology nurses	n=15; SIT	n=15; Attention control	Stand-alone mobile app on mobile phone	8 videos over 4 weeks; no support	STAI (anxiety trait): *d*=0.41^a,d,f^ COPE (Active): *d*=-0.45^a,d,f^ COPE (Denial): *d*=0.53^a,d,f^
Grassi et al (2011); *Mobile Stress Management*	RCT	Stress	Female university students	n=not reported; SIT	n=not reported; Control	Stand-alone mobile app on mobile phone	6 videos over 6 days; no support	NA
Watts et al (2013); *Get Happy Program*	Pilot RCT	MDD	Adults from the community	n=15; CBT via mobile app	n=20; CBT via computer	Stand-alone mobile app on mobile phone + iPad	6 modules over 8 weeks; MHP	PHQ-9: *d*=1.56^a,e,f^ *d*=-0.14^a,g^ *d*=1.69^b,e,f^ *d*=-0.28^b,g^ BDI-II : *d*=1.90^a,e,f^ *d*=-0.11^a,g^ *d*=2.11^b,e,f^ *d*=-0.48^b,g^ K10: *d*=1.93^a,e,f^ *d*=0.01^a,g^ *d*=1.23^b,e,f^ *d*=0.03^b,g^

^a^posttest

^b^follow-up

^c^within immediate coaching session

^d^ *P*<.05

^e^ *P*<.001

^f^within-group effect size;

^g^between-group effect size

### Quality Assessment

The quality of the studies varied but was generally low (see Table 2). Three studies describing 2 apps reported adequate sequence generation [21,22,25], whereas 3 studies [19,20,24] did not outline their sequence generation method. Three studies [21,22,25] reported allocation to conditions by an independent (third) party, whereas 3 other studies [19,20,24] did not provide sufficient information on allocation. Two studies that included diagnostic interviews [21,22] reported using blinded outcome assessors, and 4 studies [19,20,24,25] did not report blinding of assessors or used self-report outcome measures. Two studies [11,23] were not eligible for ratings for sequence generation, allocation concealment, or blinding of outcome assessors due to the pre-post study design. In 6 studies [11,21-25], ITT analyses (completeness of follow-up data) were conducted; 1 of these failed to describe dropout rates [23], and only 1 study [11] described reasons for dropout during the intervention. Two studies [19,20] did not state the nature of the statistical analyses or dropout rate at all. Insufficient information and a high risk of bias of selective outcome reporting was present in 3 studies [19,20,23] and 2 studies [21,22] respectively. Three studies [11,23,25] had a high risk of other sources of bias (eg, absence of a control group, possible treatment infidelity) while for 5 studies [19-22,24] the risk of bias from other sources was unclear (due to significant difference at baseline for stress outcome, unequal number of participants in intervention and control group, and insufficient information). None of the included studies met all 6 quality criteria of the Cochrane tool (see Table 2).

**Table 2:** Risk of bias assessed by Cochrane Risk of Bias Tool^a^.

Trials	Sequence generation	Allocation concealment	Blinding	Incomplete outcome data	Selective outcome reporting	Other sources of bias	Total
Burns et al, 2011	NA	NA	NA	0	0	2	2
Grassi et al, 2011	1	1	1	1	1	1	6
Kauer et al, 2012	0	0	0	1	2	1	4
Reid et al, 2011	0	0	0	1	2	1	4
Rizvi et al, 2011	NA	NA	NA	1	1	2	4
Villani et al, 2011	1	1	1	1	0	1	5
Villani et al, 2012	1	1	1	1	1	1	6
Watts et al, 2013	0	0	1	0	0	2	3

^a^0: low risk of bias; 1: insufficient information; 2: high risk of bias; NA: not applicable.

### Effects of the Mental Health Apps

#### Depression

Four studies describing 3 mobile apps [11,21,22,25] targeted depression. Burns et al [11] found a significant reduction in depression caseness (Mini-International Neuropsychiatric Interview [MINI]: Z=2.15, beta [week]=-.65, *P*=.03), as well as depression and anxiety symptoms at posttest (Patient Health Questionnaire [PHQ-9]: *d*=1.95, *P*<.001; Quick Inventory of Depression Symptoms-Clinician Rated: *d*=2.28, *P*<.001; Generalized Anxiety Disorder-7 item scale: *d*=1.37, *P*<.001) in a pilot test of the guided Mobilyze! app alongside a website and EMA for adults from the general population. The Mobilyze! app will be publicly available for download soon.

In a randomized controlled trial (RCT) of a guided mobiletype app with EMA conducted by Kauer et al [21] and Reid et al [22], no significant differences were found at posttest and follow-up on outcomes of depression, anxiety, and stress among adolescents from general practice compared to an attention control group (Depression and Anxiety Stress Scale [DASS] anxiety: *d*=0.07, *P*=.76; DASS depression: *d*=0.09, *P*=.69). However, it should be noted that the control group received largely the same intervention as the experimental group, with the exception of two components; ESA training via EMA and minimal feedback reports. Mediator analyses yielded an indirect effect of group on depression via ESA (beta=–0.610, 95% CI –5.596 to –0.003). Significant small to moderate within-group differences over time were found for the intervention group (DASS stress: *d*=0.37 at posttest; *d*=0.59 at follow-up; DASS anxiety: *d*=0.31 at posttest; *d*=0.45 at follow-up; DASS depression: *d*=0.34 at posttest; *d*=0.64 at follow-up) and control group (DASS stress: *d*=0.14 at posttest; *d*=0.41 at follow-up; DASS anxiety: *d*=0.07 at posttest; *d*=0.08 at follow-up; DASS depression: *d*=0.42 at posttest; *d*=0.61 at follow-up). Between-group effect sizes were small and nonsignificant (DASS stress: *d*=0.14 at posttest; *d*=0.22 at follow-up; DASS Anxiety: *d*=0.25 at posttest; *d*=0.07 at follow-up; DASS depression: *d*=0.11 at posttest; *d*=0.09 at follow-up). The mobiletype app is not publicly available for download to date.

Watts et al [25] found a significant reduction over time (*P*<.001) and large effect sizes in a pilot RCT of a partially guided CBT-based program for depression delivered either via a computer or mobile app (Get Happy Program) (PHQ-9: *d*=1.56; Beck Depression Inventory [BDI-II]: *d*=1.90; Kessler10 [K10]: *d*=1.93). No differences between the two groups were found for depression over time with *P*>.05 (PHQ-9: *P*=.34, *d*=–0.14; BDI-II: *P*=.52, *d*=–0.11; K10: *P*=.90, *d*=–0.01). The Get Happy app is not publicly available for download to date.

#### Anxiety/Stress

Three RCTs describing 1 unguided mobile app (Mobile Stress Management) using SIT [19,20,24] found a significant decrease in state and trait anxiety (State and Trait Anxiety Inventory [STAI]) and a significant increase in active coping skills among oncology nurses [20,24] and female university students [19] compared to a control group. Grassi et al [19] used a simplified version of the Mobile Stress Management app, which was also effective for reducing stress. However, both Villani et al [20] and Grassi et al [19] did not provide statistical results for intervention versus control group comparisons. Villani et al [24] reported significant decreases in state anxiety over time (*F*_1,28_=71.365, *P*≤.001) and a significant *group x time* interaction effect for state anxiety (*F*_1,28_=27.476, *P*≤.001). Within-group effect sizes (converted from *t* test statistics) were small for active coping strategies (COPE Inventory [COPE] Active: *d*=0.42), and large for state anxiety (STAI: *d*=0.84) and denial coping strategies (COPE Denial: *d*=1.08). The Mobile Stress Management app is publicly available for download (Italian version only).

#### Substance Use

A pilot feasibility study aiming to reduce substance use (alcohol, drugs, and tobacco) among adults suffering from borderline personality disorder using a mobile app (DBT Coach [23]) in conjunction with face-to-face DBT therapy, indicated a significant reduction (*P*<.05) within each DBT Coach session in emotional intensity and urge to use substances (*d*=0.52 and *d*=0.29 respectively). Furthermore, a significant reduction (*P*<.05) in symptoms of depression (BDI: *P*=.014, *d*=0.55), global symptom severity (Brief Symptom Inventory: *P*=.021, *d=*0.43), and confidence in participants’ ability to use opposite action (ie, emotion regulation) skills (Behavior Confidence Questionnaire: *P*=.008, *d*=0.59) was noted from pre- to post assessment. Multimedia Appendix 5 outlines the ITT within-group effect sizes. The DBT Coach app is publicly available for download.

#### Ecological Momentary Assessment

Mixed findings were obtained from the 2 studies using EMA as part of the intervention. In the Burns et al [11] study, promising accuracy rates (60-91%) were achieved in predicting categorical contextual states (eg, location) based upon participant EMA entries. For participant states rated on continuous self-report scales (eg, mood), predictive capability was poor. Notwithstanding these technological outcomes, Reid et al [21] and Kauer et al [22] demonstrated that increased self-monitoring with EMA by participants did lead to increased ESA and thereby reduced depressive symptoms.

#### Intervention Feasibility and Adherence

Three studies providing usability and feasibility outcomes (eg, acceptability of the technology, perceived usefulness, perceived utility) reported moderate to high rates of mobile phone usage, feasibility, and participant satisfaction with the intervention [11,23,25]. The dropout rate was reported in 4 studies and varied between 12.5% and 34.3% [11,21,22,25]. Reported reasons for dropout, where described, were mostly due to technical problems [11].

## Discussion

### Principal Results and Comparison With Prior Work

In general, the studies included in this systematic review showed promising results for evidence-based mental health apps in reducing depressive symptoms and caseness, stress, anxiety, and substance use, similar to previous reviews of mHealth [6,7]. However, due to the high risk of bias in some studies, these findings need to be considered with caution pending replication. Due to the absence of a control group in 2 studies [11,23], it was difficult to determine whether the beneficial effects were attributable to the app itself, a function of natural remission or regression to the mean, or in case of the DBT Coach app [23], due to the face-to-face DBT therapy offered to all participants in conjunction with the app. Additionally, a clear conclusion about the efficacy of the DBT Coach for substance use treatment cannot be drawn yet, since—besides the absence of a control group—change in substance use (eg, amount of alcohol units per week) prior to or after treatment was not reported, nor was a distinction made between different types of substance use (alcohol, drugs, nicotine cessation). Furthermore, some studies failed to provide sufficient information regarding dropout rates or did not report the statistical analyses used [19,20].

The mobiletype app was the only intervention that failed to yield any significant direct effect on depression, although a significant indirect effect was found in a reduction of depressive symptoms through the direct effect of increased ESA [21]. Because the attention-placebo control group received almost the same intervention as the experimental group, except for the ESA component, the nonsignificant finding is likely to be the cause of this finding. This study suggests that repeated self-monitoring over time using EMA on a mobile device may increase ESA and thereby reduce depressive symptoms. Evidence supports a similar mechanism underlying improvements in depression with CBT, where one of the most important components of CBT for depression involves rating one’s mood and activities in a diary to raise awareness of how activities influence mood states [26]. The development of mobile devices has facilitated the collection of EMA data, thereby providing a portable and convenient delivery mode with which an individual can incorporate EMA and regular mood monitoring in their daily lives and improve ESA as part of treatment for depression. Although EMA shows promising results in predicting categorical contextual states, it needs to be further optimized to be able to accurately predict mood states [11]. Once refined in such a way to maximize accuracy and temporal resolution and minimize bias, EMA holds considerable potential to reveal dynamic interplay between mood, cognition, and behavior, increase participant self-awareness of such processes, and thereby enhance mental health treatment [27]. Together with the use of biomedical and/or activity sensors, timely personalized feedback can be generated to prompt users. mHealth interventions therefore have the potential to improve current depression treatment considerably [10]. In a similar way to guided Internet interventions [28], guided apps might derive larger effect sizes and adherence rates than stand-alone self-help apps, but more research is necessary to elucidate this.

### Usability, Helpfulness, and Satisfaction

Usability, helpfulness, and satisfaction ratings, where assessed, were moderate to high [11,23,25], indicating that mHealth apps are perceived to be a useful vehicle for enhancing access to evidence-based monitoring and self-help. However, common technical problems (eg, battery failure, connectivity, freezing of app) need to be overcome. Adherence rates (if reported) were high, in line with previous research in mHealth [29], but higher when compared to adherence rates seen with Internet-based interventions [30]. It might be that the method of delivery (mobile phone) and its portability and flexible usage, and/or its delivery of personalized feedback may account for these higher retention rates for mobile apps. However, some of the included studies provided subjects with monetary rewards for participation, which is likely to artificially raise adherence rates as well.

### Sustainability of Results

Most studies included only posttest assessment or a short-term follow-up (6 weeks). Although 1 study showed sustainable results at 3-month follow-up [25], sustainability of results over a medium- to long-term timeframe requires further investigation and replication. As such, on the basis of current evidence, sustainability of results cannot yet be determined.

Since mental health apps downloadable for use by the general population are increasing rapidly, despite evidence for their efficacy being largely unknown, the focus of this systematic review was on apps only. We applied very stringent inclusion criteria to ensure that we identified the evidence-based mental health apps that could be downloadable in the future by the general public from app stores, for example, Google Play for Google Android [14] or the Apple App Store [15]. Therefore, several highly sophisticated programs using mobile technology were excluded, such as the myCompass program [31] for depression, anxiety, and stress. The CBT-based myCompass program is delivered via a website with an Internet-enabled mobile phone component and encourages real-time self-monitoring of moods, mood triggers, and lifestyle behaviors using SMS text messaging and email prompts. Other examples of similar programs include an SMS-based txt2quit intervention [32] and a video-based STUB IT intervention [33], both of which have been shown to be effective for smoking cessation, and an SMS-based intervention [34] to increase medication adherence in individuals with schizophrenia. We were also unable to include the innovative INTREPID research [35], which used virtual reality exposure therapy on mobile phones to reduce anxiety.

There are more than 3000 mental health apps for Android, Apple, and Microsoft freely available to download to date, compared to the 8 evidence-based apps we identified through our systematic review. Only 2 of the apps included in this review are currently available for public download, comprising less than 1% of the commercially available apps. A recently published review on existing (commercial) mHealth apps for the most prevalent health conditions in the Global Burden of Disease list provided by the World Health Organization [36] echoes this finding. The authors concluded that the development of mHealth apps was first and foremost driven by commercial and economic motivations rather than scientific motivations behind research. Although the numerous protocols [9,37] and case studies [38,39] we excluded indicate a nascent field of research, the rapid growth and development of thousands of non-evidence-based mental health technologies has generated the need for independent regulation. This is underlined by the alarming findings from previous research [40-42] indicating that only 13-26% of Web-based or app-based interventions for smoking cessation adhere to treatment guidelines. A recent study on commercial apps using EMA for alcohol use echoes these findings [43]. The US Food and Drug Administration has taken an important step towards the development of quality control guidelines for health apps [44], but there are still major issues and dangers concerning the lack of quality control of commercially available mental health apps. Further research and work must be undertaken to develop, test, and disseminate evidence-based mHealth interventions among the public to ensure optimal public health outcomes.

### Limitations

This review has several limitations. First, despite an extensive search, the number of included studies was small, which restricted our interpretations as to whether mHealth apps have an effect on reducing mental health symptoms. Second, the number of participants in the included studies was small. As a result, the studies were probably underpowered to detect the more subtle effects of the interventions. Furthermore, small sample sizes hamper the precision and accuracy of the statistical results and therefore limit our interpretations [45]. Third, the quality of the included studies was low. Historically, low quality trials yield positive results [46]. Due to the small number of studies, we were unable to examine whether significant differences existed between higher- and lower-quality studies. Fourth, there were no studies that examined the long-term efficacy of mental health apps. Therefore, long-term effects remain as yet unknown. Finally, only studies from peer-reviewed, English language journals were included in this review. However, the effect of language bias has been shown to have a minimal impact on the conclusions of systematic reviews [47].

### Future Research

There is a very clear need for more research in this area. Trials with an RCT design of high quality to minimize risk of bias are needed to determine the efficacy of mental health apps. Unfortunately, the competitive nature and time-consuming process of grant applications and RCT designs necessary for such high-quality research contrasts sharply with the speed of development in this highly innovative technology. Component testing with small sample sizes may offer one solution to help bridge the gap between academia and real-world applications [48]. Research is particularly weak in the domains of sleep disturbance, anxiety disorders, and smoking cessation and needs further investigation. The cost-effectiveness and cost-utility of mHealth, compared to standard care or Internet-based treatment, requires further examination.

### Conclusions

In summary, although a firm conclusion cannot yet be drawn, the current systematic review suggests that mobile apps for mental health have the potential to be effective in reducing depression, anxiety, stress, and possibly substance use for individuals experiencing these symptoms. Given the widespread usage of mobile and smartphones and increasing uptake of tablet devices, mHealth has the potential to increase treatment accessibility globally. The difference in the volume of commercial apps compared to the small number of tested evidence-based apps is striking. It warrants the need for public education and further development and research into evidence-based mental health apps and consideration of industry regulation.

## Multimedia Appendix 1

Search string MEDLINE and Embase.

## Multimedia Appendix 2

Search string Cochrane Compendex and Inspec.

## Multimedia Appendix 3

Search string PsycINFO and PsycTESTS.

## Multimedia Appendix 4

Individual author names.

## Multimedia Appendix 5

Psychosocial studies of applications on mobile devices (intention-to-treat).

## Abbreviations

BA: behavioral activation
BDI: Beck Depression Inventory
BDI-II: Beck Depression Inventory-Second edition
CBT: cognitive behavior therapy
COPE: COPE Inventory
CT: cognitive therapy
DASS: Depression, Anxiety and Stress Scale
DBT: dialectical behavior therapy
EMA: ecological momentary assessment
ESA: emotional self-awareness
ITT: intention-to-treat
K10: Kessler Psychological Distress Scale-10 item scale
MDD: major depressive disorder
MINI: Mini-International Neuropsychiatric Interview
PHQ: Patient Health Questionnaire
PHQ-9: Patient Health Questionnaire-9 item scale
RCT: randomized controlled trial
SIT: stress inoculation training
STAI: State-Trait Anxiety Inventory
