# Supplementary figures and images for: Smartphones for Smarter Delivery of Mental Health Programs: A Systematic Review
Source: J Med Internet Res. 2013 Nov 15;15(11):e247. doi: 10.2196/jmir.2791 (PMC3841358; doi:10.2196/jmir.2791)

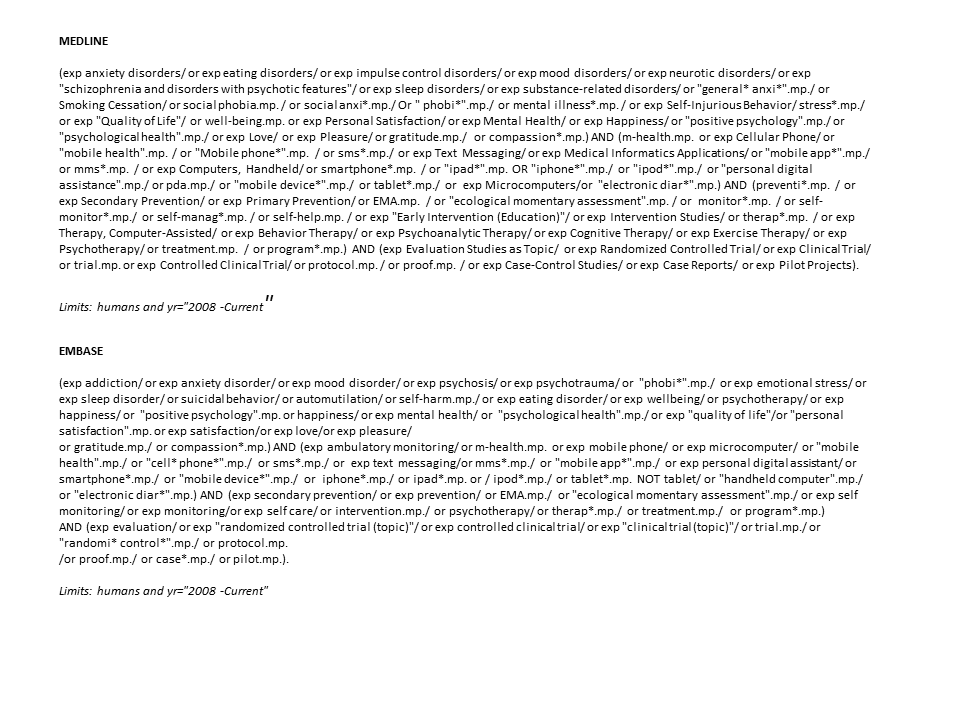

Supplement: Supplementary file 1 [file jmir_v15i11e247_app1.GIF]

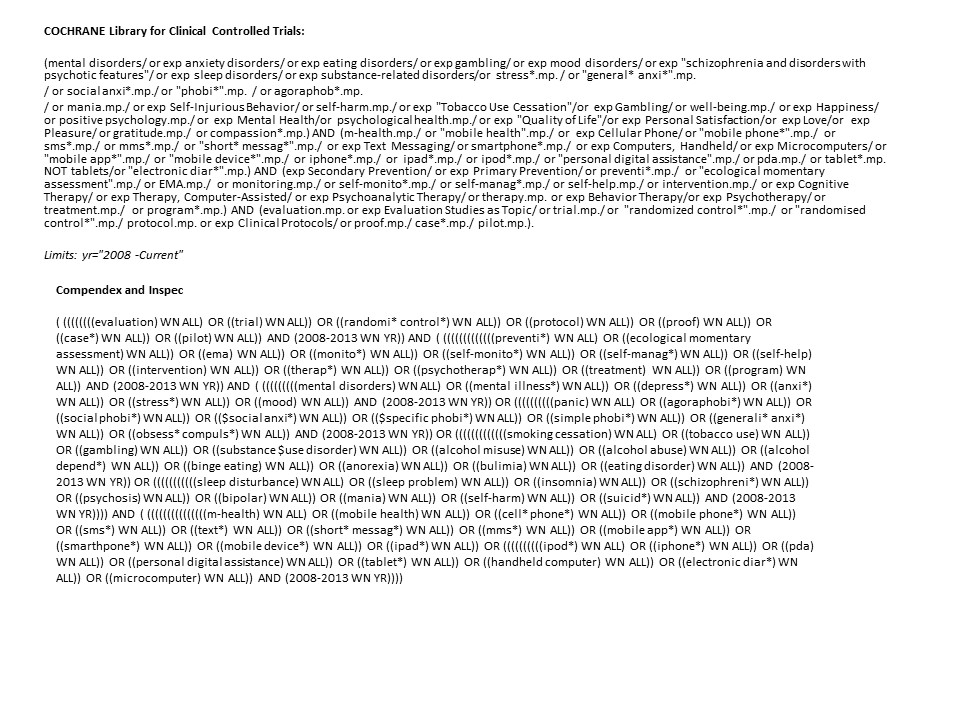

Supplement: Supplementary file 2 [file jmir_v15i11e247_app2.GIF]

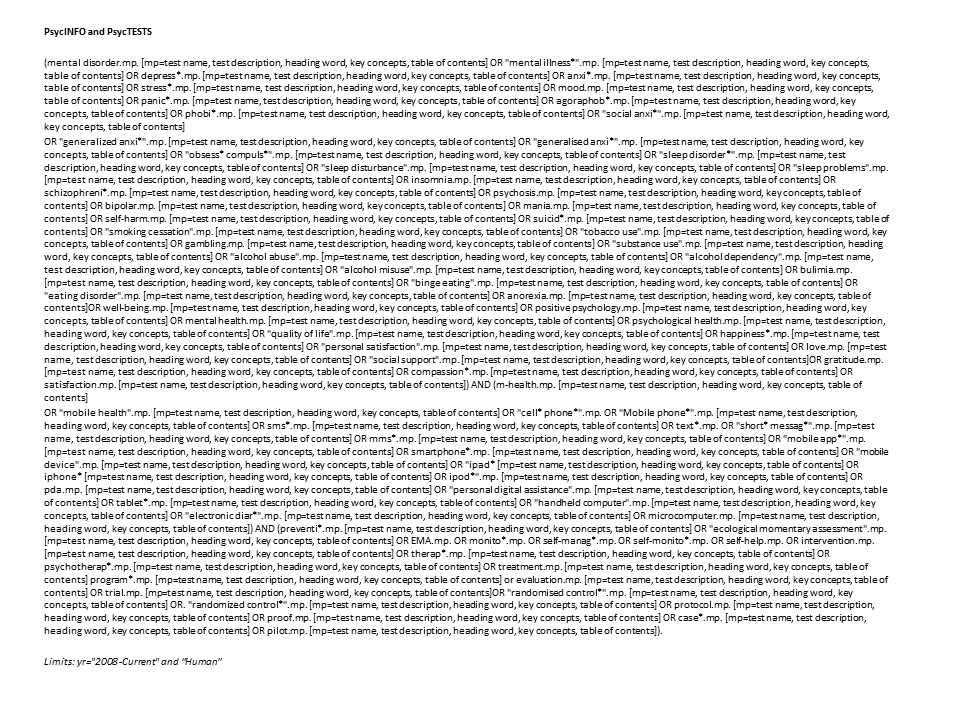

Supplement: Supplementary file 3 [file jmir_v15i11e247_app3.GIF]
