# Supplementary material for: Smartphones for Smarter Delivery of Mental Health Programs: A Systematic Review
Source: J Med Internet Res. 2013 Nov 15;15(11):e247. doi: 10.2196/jmir.2791 (PMC3841358; doi:10.2196/jmir.2791)
Supplement: Supplementary file 4 [file jmir_v15i11e247_app4.pdf]

Multimedia Appendix 4: Search on prominent individual authors' and researchers' names in the field of mHealth or internet interventions in Medline

Anderson, G.

Andrews, G.

Ben-zeev, D.

Bockting, E.

Botella, C.

Both, F.

Burns, M.

Carlbring, P.

Cuijpers, P.

Grassi, A.

Griffiths, K.M.

Mohr, D.

Morris, M.E.

Munoz, R.

Kauer, S.D.

Reid, S.C.

Riper, H.

Ritterband, L.

Riva, G.

Rizvi, S.

Vilanni, D.

Warmerdam, E.H.

Watts, S.

Whittaker, R.
