# Supplementary material for: Smartphones for Smarter Delivery of Mental Health Programs: A Systematic Review
Source: J Med Internet Res. 2013 Nov 15;15(11):e247. doi: 10.2196/jmir.2791 (PMC3841358; doi:10.2196/jmir.2791)
Supplement: Supplementary file 5 [file jmir_v15i11e247_app5.pdf]

Multimedia Appendix 5. Psychosocial studies of applications on mobile devices (intention-to-treat).

**Author (year); name of app**	**Trial**	**Primary outcome measure**	**Study sample**	**Intervention group**	**Control group**	**Delivery type**	**Delivery length and support**	**Drop-out rate**	**Assessment/ period(s)**	**Within[7] and between[8] effect size (Cohen's *d*)**	**Pub DL?[4]**
Burns et al (2011);<br><br>*Mobilyze!*	Pre-post pilot	MDD	Adults from the community	n=8; BA	N.A.	Mobile app + website + EMA on mobile phone	8 weeks; MHP	12.5%	PHQ, QIDS-C, GAD-7/ Post-test only	PHQ-9: *d*=1.95[1,6,7]<br>QIDSC: *d*=2.28[1,6,7]<br>GAD-7: *d*=1.37[1,6,7]	No
Kauer et al (2012);<br>Reid et al (2011);<br><br>*Mobile-type*	RCT	MDD	Adolescents from general practice	n=68; ESA + Individua-lized data summary reports + meeting with GP	n=49; Attention Control + Part of the individualized data summary reports + meeting with GP	Stand-alone mobile app + EMA on mobile phone	8 modules over 2-4 weeks; MHP	22.3%	DASS, ESA/ Post-test and 6 weeks follow-up	DASS Stress:<br>*d*=0.37[1,7]<br>*d*= 0.59[2,6,7]<br>*d*=0.14[1,8]<br>*d*=0.22[2,8]<br>DASS Anxiety:<br>*d*=0.31[1,7] *d*=0.45[2,5,7]<br>*d*=0.25[1,8]<br>*d*=0.07[2,8]<br>DASS Depression:<br>*d*=0.34; *d*=0.64[2,6]<br>*d*=0.11[1,8]<br>*d*=0.09[2,8]<br>ESA:<br>*d*=0.31[1]; *d*=0.66[2,5]<br>*d*=0.09[1,6,8]<br>*d*=0.58[2,5,8]	No
Rizvi et al (2011);<br><br>*DBT Coach*	Pre-post pilot	BPD and substance use	Adults from out-patient clinic	N=21; DBT + OA	N.A.	Mobile app on mobile phone + F2F DBT	10-14 days; MHP	N.A.	BDI, BSI, DBT Coach Data, BCQ/ Post-test only	BDI: *d*=0.55[1,5,7]<br>BSI: *d*=0.43[1,5,7]<br>BCQ: *d*=0.59[1,5,7]<br>Emotional intensity to use substance: *d*=0.52[3, 5,7]<br>Urge to use substance: *d*=0.29[3, 5,7]	Yes
Villani et al (2012);	RCT	Stress	Female oncology nurses	n=8; SIT	n=8; Attention Control	Stand-alone mobile app	8 videos over 4	N.A.	STAI, COPE/ Post-test only	N.A.	Yes (It)

Mobile Stress Management						on mobile phone	weeks; no support				
Villani et al (2011);<br><br>*Mobile Stress Management*	RCT	Stress	Female oncology nurses	n=15; SIT	n=15; Attention Control	Stand-alone mobile app on mobile phone	8 videos over 4 weeks; no support	N.A.	STAI, COPE/ Post-test only	STAI (anxiety trait): *d*=0.41[1,5,7]<br>COPE (Active): *d*=-0.45[1,5,7]<br>COPE (Denial): *d*=0.53[1,5,7]	Yes (It)
Grassi et al (2011);<br><br>*Mobile Stress Management*	RCT	Stress	Female university students	n=not reported; SIT	n= not reported; Control	Stand-alone mobile app on mobile phone	6 videos over 6 days; no support	N.A.	STAI, COPE/ Post-test only	N.A.	Yes (It)
Watts et al (2013);<br><br>*Get Happy Program*	Pilot RCT	MDD	Adults from the community	n=15; CBT via mobile app	n=20; CBT via computer	Stand-alone mobile app on mobile phone + iPad	6 modules over 8 weeks; MHP	31.4%	PHQ-9, BDI-II, K10/ Post-test and 3 months follow-up	PHQ-9: *d*=1.56[1,6,7]<br>*d*=-0.14[1,8]<br>*d*=1.69[2,6,7]<br>*d*=-0.28[2,8]<br>BDI-II : *d*=1.90[1,6,7]<br>*d*=-0.11[1,8]<br>*d*=2.11[2,6,7]<br>*d*=-0.48[2,8]<br>K10:<br>*d*=1.93[1,6,7]<br>*d*=0.01[1,8]<br>*d*=1.23[2,6,7]<br>*d*=0.03[2,8]	No

App: Application;
BA: Behavioral Activation;
BCQ: Behavior Confidence Questionnaire;
BDI: Beck Depression Inventory;
BPD: Borderline Personality Disorder;
BSI: Brief Symptom Inventory;
COPE: COPE Inventory;
DASS: Depression, Anxiety and Stress Scale;

DBT: Dialectical Behavior Therapy;
ESA: Emotional Self Awareness;
F2F: Face-to-Face;
GAD-7: Generalized Anxiety Disorder-7 item scale;
GP: General Practitioner;
It: Italian;
K10: Kessler Psychological Distress Scale-10 item scale;
MDD: Major Depressive Disorder;
MHP: Mental Health Professional (psychologist or psychotherapist, GP);
MINI: Mini-International Neuropsychiatric Interview;
N.A.: Not Applicable;
OA: Opposite Action;
PHQ: Patient Health Questionnaire;
QIDS-C: Quick Inventory of Depression Symptoms-Clinician Rated;
RCT: Randomized Controlled Trial;
SIT: Stress Inoculation Training;
STAI: State-Trait Anxiety Inventory.
[1]posttest; [2]follow-up; [3]within immediate coaching session; [4]publicly available for download; [5]$P<.05$; [6]$P<.001$; [7]within-group effect size; [8]between-group effect size
